# Histologic Features and Immunodetection of African Tick-bite Fever Eschar

**DOI:** 10.3201/eid1209.051540

**Published:** 2006-09

**Authors:** Hubert Lepidi, Pierre-Edouard Fournier, Didier Raoult

**Affiliations:** * Université de la Méditerranée, Marseille, France

**Keywords:** African tick-bite fever, “tâche noire,” eschar, *Rickettsia africae*, cutaneous biopsy, pathology, immunohistochemistry, research

## Abstract

African tick-bite fever (ATBF) is a rickettsiosis caused by *Rickettsia africae*. We describe histologic features and immunodetection of *R. africae* in cutaneous inoculation eschars from 8 patients with ATBF, which was diagnosed by culture or association of positive PCR detection and positive serologic results. We used quantitative image analysis to compare the pattern of inflammation of these eschars with those from Mediterranean spotted fever. We evaluated the diagnostic value of immunohistochemical techniques by using a monoclonal antibody to *R. africae*. ATBF eschars were histologically characterized by inflammation of vessels composed mainly of significantly more polymorphonuclear leukocytes than are found in cases of Mediterranean spotted fever (p<0.05). Small amounts *R. africae* antigens were demonstrated by immunohistochemical examination in 6 of 8 patients with ATBF. Neutrophils in ATBF are a notable component of the host reaction, perhaps because ATBF is a milder disease than the other rickettsioses. Immunohistochemical detection of rickettsial antigens may be useful in diagnosing ATBF.

## Abstract 2

Immunohistochemical detection of rickettsial antigens may be useful in diagnosis.

African tick-bite fever (ATBF), a recently rediscovered rickettsiosis of the spotted fever group, is caused by *Rickettsia africae*, an obligate intracellular, gram-negative, rod-shaped bacterium, transmitted by cattle ticks of the *Amblyomma* genus (*1**–**5*). ATBF is highly prevalent in Africa and often affects visitors to this region (*1**,**6**–**8*). In patients, ATBF manifests as an acute, febrile, and influenzalike illness, frequently accompanied by severe headache, prominent neck muscle myalgia, inoculation eschars (which appear as black crusts surrounded by a red halo at the site of the tick bite), and regional lymphadenitis (*5*). As many as 50% of patients have multiple eschars (*1*).

Inoculation eschars occur at the sites of tick bites and consist of a focus of epidermal and dermal necrosis ≈1 cm in diameter; they represent the portal of entry of the infectious agent into the host and the first site of challenge between the infected human host and the bacterium. Eschars have been reported and their pathologic features have been described in other spotted fever rickettsioses such as Mediterranean spotted fever, North Asian tick typhus, or Queensland tick typhus (*9**–**11*). Despite several clinical descriptions of ATBF, the pathologic features of the inoculation eschar have not yet been described. To determine the pathologic characteristics associated with *R. africae* infection at the cutaneous level, we analyzed skin biopsy specimens of patients with confirmed ATBF and compared the results with those from patients with Mediterranean spotted fever, the rickettsiosis caused by *R. conorii*, with respect to the pattern of inflammatory reaction. Moreover, we performed immunohistologic testing for localization of the bacteria in skin biopsy specimens. The specific detection of *R. africae* may be useful when ATBF is suspected, especially in differentiating this rickettsiosis from other spotted fever group rickettsioses.

## Patients, Materials, and Methods

### Case Definition

Patients referred to our laboratory from 1999 to 2004 were classified as definite cases of *R. africae* infection if this rickettsia was isolated from clinical specimens or a positive PCR detection was associated with a positive serologic test result. Of these, we selected those for whom inoculation eschar biopsy specimens had been formalin fixed and paraffin embedded for histopathologic analysis.

### Serology, PCR, and Culture

Immunofluorescence assays, the reference diagnostic method, were carried out by using both *R. conorii* strain Seven (Malish, ATCC VR-613T) and *R. africae* strain ESF-5 as antigens (*6*). Titers of 64 for IgG and 32 for IgM in patient serum specimens were considered evidence of recent infection by a *Rickettsia* sp (*12**–**14*). Western blotting and cross-absorption procedures were performed (*15**–**17*). The microorganism was isolated in the manner previously reported (*6*). DNA was extracted from material frozen at –80°C. DNA was extracted from ground eschar biopsy specimens by using the QIAmp Tissue kit (Qiagen GmbH, Hilden, Germany), according to the manufacturer's recommendations. These DNA extracts were used as templates in a PCR assay incorporating the *ompA* primers 190-70 and 190-701 (*18*). For negative controls, we used sterile water processed as described and DNA extracted from a heart valve from a patient with degenerative valvulopathy. For positive controls, we used DNA from *R. montanensis* strain M/5-6. Testing was performed in a blinded manner. All positive PCR products were sequenced in both directions (*19*). When regular PCR did not detect rickettsiae in skin biopsy specimens, we used a nested PCR incorporating the *omp*A-amplifying primer sets AF1F–AF1R and then AF2F–AF2R for the nested amplification (*1*). Every 6 specimens, we incorporated the above-described negative controls. To avoid contamination, we did not include any positive control in the assay. Positive nested PCR products were identified by sequencing with the AF2F–AF2R primer pair.

### Histologic Analysis and Immunohistochemical Detection of *R. africae*

Formalin-fixed, paraffin-embedded skin biopsy specimens of the inoculation eschars were cut to 3 μm in thickness and stained with hematoxylin-eosin-saffron by routine staining methods. Serial sections of each tissue specimen were also obtained for special staining or immunohistochemical investigations. Immunohistochemical analysis was performed on paraffin-embedded skin biopsy sections by use of the monoclonal mouse antibody AF8-F3 produced against *R. africae* (*20*). The specificity of this antibody has been tested on a variety of rickettsiae, and cross-reactivity was found only with *R. parkeri* and *R. sibirica* (*20*). The immunohistologic procedure, in which an immunoperoxidase kit was used, has been described elsewhere (*21*). Briefly, after deparaffinization, each tissue section was incubated with the monoclonal anti–*R. africae* antibody diluted 1:1000 in phosphate-buffered saline. After the sections were incubated with the primary antibody, immunodetection was performed with biotinylated immunoglobulins, followed by peroxidase-labeled streptavidin (HistoStain plus kit, Zymed, Montrouge, France) with amino-ethyl-carbazole as substrate. The slides were counterstained with Mayer hematoxylin for 10 min. For each case, 3 level tissue sections were systematically evaluated by immunohistochemical analysis, and a negative control was created by using an irrelevant monoclonal mouse antibody. Moreover, to test the specificity of our monoclonal antibody, skin biopsy specimens from patients with Mediterranean spotted fever (15 cases), acute eczematous dermatitis (2 cases), psoriasis (2 cases), and lichen planus (1 case) served as negative controls.

### Quantitative Image and Statistical Analyses

To characterize the immune response in inoculation eschars during ATBF and Mediterranean spotted fever, paraffin sections were stained with the polymorphonuclear leukocyte marker CD15 (Immunotech, Marseille, France), the macrophage marker CD68 (Dako, Trappes, France), the T-lymphocyte marker CD3 (Dako), the B-lymphocyte marker CD20 (L26, Dako), and the endothelial cell marker Factor VIII–related antigen (Dako) by using the peroxidase-based method described above. The antibodies anti-CD15 and anti-CD3 were ready to use, whereas the antibodies anti-CD68, anti-CD20, and anti-Factor VIII–related antigen were used at a working dilution of 1:1000.

The evaluation of the proportion of polymorphonuclear leukocytes, macrophages, T lymphocytes, and B lymphocytes, as well the relative proportion of neovascularization in skin tissue specimens, was determined by quantitative image analysis (*22*). In brief, histologic images were digitized and transferred to a computer system. Using the image analyzer SAMBA 2005 (SAMBA Technologies), which is a specific interactive program that provides visual control of analysis, we analyzed the CD15-positive, CD68-positive, CD3-positive, CD20-positive, and Factor VIII–positive surfaces in tissue sections to determine the percentages of the total surface area covered by neutrophils, macrophages, T and B lymphocytes, and endothelial cells, respectively. For each set of observations, the surfaces of 10 randomly chosen areas were studied at a magnification ×100, and the surface areas of each immunohistologic parameter were measured. The average areas were calculated by comparison with the area of the whole tissue sample. The Mann-Whitney U test was used for the statistical comparisons of values that were obtained for each immunohistologic parameter for skin biopsy specimens from patients with ATBF and patients with Mediterranean spotted fever; p<0.05 was considered significant.

### Value of Immunohistochemical Techniques

The sensitivity, specificity, and positive and negative predictive values of immunohistochemical techniques for the diagnosis of ATBF were calculated. The sensitivity of immunohistochemical analysis was compared to that of culture, regular PCR, nested PCR, and serologic testing by using the Fisher exact test.

## Results

Skin biopsy specimens from 8 patients with ATBF, from 1999 to 2004, obtained from black crusts corresponding to inoculation eschars, were positive for *R. africae* by culture (4 patients) or PCR and serologic tests (4 patients). The age of the eschars at the time of biopsy varied from 5 to 10 days. The epidemiologic and clinical features of the cases in the 8 patients with ATBF are summarized in Table 1. The mean age of patients was 46.87 years (standard deviation 13.17 years, range 27–63 years); 4 were men and 4 were women. A history of tick bite was reported by 3 (37.5%) of 8 patients. Clinical features included fever in 6 (75%) of 8 patients, a vesicular cutaneous rash in 5 (62.5%) of 8 patients, regional lymphadenopathies in 3 (42.85%) of 7 patients, and headache and myalgia in 5 (71.42%) of 7 patients. The inoculation eschar was single in 3 (37.5%) of 8 patients and multiple in 5 (62.5%) of 8 patients. Erythema and swelling surrounded the eschar in 7 (87.5%) of 8 patients. Details of the results of laboratory tests, culture, serologic tests, PCR, and immunohistochemical tests are found in Table 2.

**Table 1:** Epidemiologic and clinical features of cases in 8 patients with African tick-bite fever

Patient no.	Age (y), sex	Country of exposure	No. eschars, site	Fever	Cutaneous rash	Regional lymphadenopathy	Headaches and myalgia
1	55, M	South Africa	>1, unknown	+	–	–	–
2	47, F	South Africa	1, leg	+	Vesicular	–	–
3	63, M	Swaziland	2, knee and shoulder	–	Vesicular	+	+
4	56, F	Swaziland	>1, unknown	+	Vesicular	+	+
5	28, F	South Africa	2, leg and chest	+	–	–	+
6	27, M	South Africa	1, leg	–	Vesicular	+	+
7	45, F	South Africa	1, calf	+	Vesicular	Unknown	Unknown
8	54, F	South Africa	3, groin and leg	+	–	–	+

**Table 2:** Biologic and pathologic features of cases in 8 patients with African tick-bite fever*

Patient no.	Culture	Serology (IFA or Western blot)	Regular PCR	Nested PCR	IHC tests
1	–	+	+	ND	+
2	+	–	+	ND	+
3	+	–	+	ND	+
4	–	–	+	ND	+
5	–	+	–	+	–
6	–	+	–	+	–
7	–	+	+	ND	+
8	+	–	+	ND	+

*IFA, immunofluorescence assay; IHC, immunohistochemical; ND, not done.

### Histologic and Immunohistochemical Analyses

**Figure 1:**
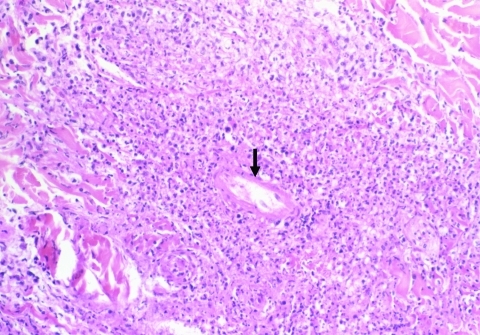
Fibrinoid necrosis of a vessel in the dermis (arrow) with perivascular inflammatory infiltrates mainly composed of polymorphonuclear leukocytes (hematoxylin-eosin-saffron; original magnification ×250).

**Figure 2:**
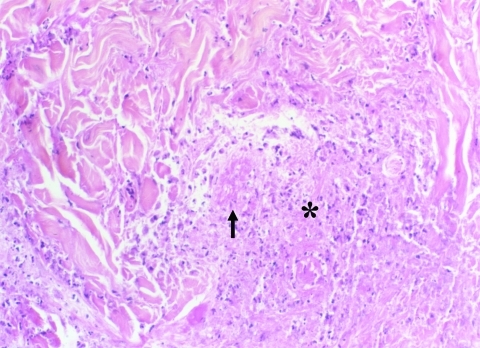
Coagulative necrosis (*) of the dermis surrounding necrotic vessels (arrow) (hematoxylin-eosin-saffron; original magnification ×250).

**Figure 3:**
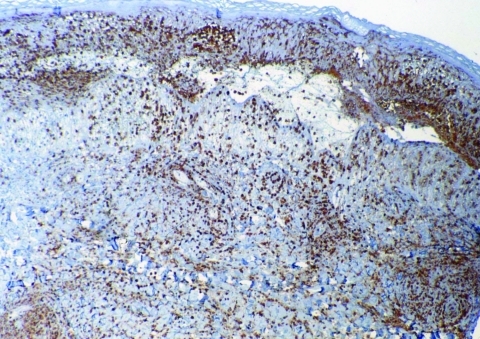
Inoculation eschar from a patient with African tick-bite fever showing numerous dermal inflammatory infiltrates mainly composed of polymorphonuclear leukocytes (immunoperoxidase staining with an anti-CD15 antibody; original magnification ×100).

**Figure 4:**
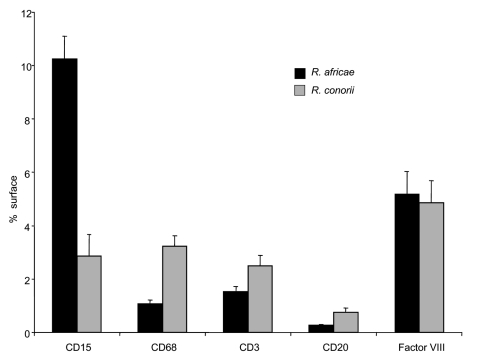
Quantification of inflammatory changes in inoculation eschars from patients with African tick-bite fever (*Rickettsia africae*, n = 8) and patients with Mediterranean spotted fever (*R. conorii*, n = 15). Surface areas expressing CD15, CD68, CD3, CD20, and Factor VIII were quantified after immunostaining. Quantification of each parameter was evaluated by computer-assisted analysis of digitized microscopic images. Results were normalized and expressed as a percentage of the total skin tissue surface area. Columns represent mean values ± standard error.

Eschars of ATBF cases were histologically dominated by vascular injury and perivasculitis, which were detected in all skin biopsy specimens examined. The host response to the vascular injury was manifested as intramural and perivascular infiltration by polymorphonuclear leukocytes, small lymphocytes, and macrophages (Figure 1). Vascular damages were endothelial swelling and vascular fibrinoid necrosis (Figure 2). Mural and occlusive fibrin thrombi were observed in a few blood vessels. Vascular injuries were associated with cutaneous necrosis (Figure 2). Moderate-to-severe cutaneous necrosis was present in 7 of 8 cutaneous biopsy specimens. When cutaneous necrosis was found, it was associated with vascular thrombosis. No hemorrhages were noted. Quantitative immunohistochemical analysis showed that the immune response in skin biopsy specimens from patients with ATBF more predominantly involved polymorphonuclear leukocytes than did specimens from patients with Mediterranean spotted fever (p<0.01, Figure 3 and Figure 4). In contrast, the inflammatory infiltrates in patients with Mediterranean spotted fever were mainly characterized by macrophages (p = 0.04, Figure 4) and T and B lymphocytes. The relative amounts of vessel formation were similar in the 2 diseases.

**Figure 5:**
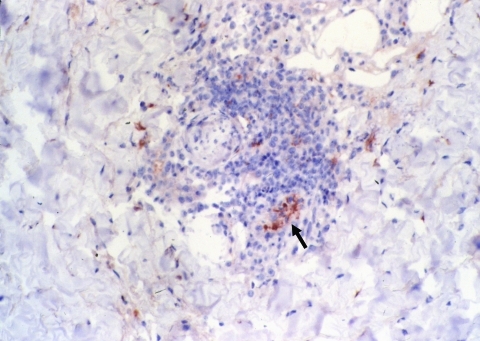
Immunohistochemical detection of *Rickettsia africae* in the inoculation eschar of a patient with African tick-bite fever. Note the location of the bacteria in the endothelial and inflammatory cells of a blood vessel in the dermis (arrow) (monoclonal rabbit anti-*R. africae* antibody used at a dilution of 1:1,000 and hematoxylin counterstain; original magnification ×250).

Six of 8 eschar biopsy specimens contained *R. africae* detected by immunohistochemical analysis (Table 2). Rickettsial antigen was observed in the endothelium and inflammatory cells organized in and around blood vessels (Figure 5). None of the control skin biopsy samples, including the *R. conorii*–infected cutaneous specimens, showed immunoreactivity with the monoclonal mouse antibody AF8-F3. Finally, no statistical correlations were found between the age of the cutaneous lesions and the amount and type of inflammatory cell infiltrate as well as the amount of rickettsial antigen found in the lesion.

The sensitivity, specificity, and positive and negative predictive values of immunohistochemistry were 75%, 100%, 100%, and 91%, respectively. The sensitivity of immunohistochemical tests was not significantly different from that of culture (6/8 vs 4/8, p = 0.6), regular PCR (6/8 vs 6/8, p = 1), nested PCR (6/8 vs 8/8, p = 0.5), and serology (6/8 vs 4/8, p = 0.6) (*1*,*12*,*28*).

## Discussion

In some rickettsioses, the inoculation eschar is the site of an intense rickettsial multiplication and thus is the preferred biopsy specimen for studying the pathologic features of *Rickettsia*-induced infection and for detecting the bacteria by immunohistologic tests as well as for carrying out isolation procedures or genomic detection. As demonstrated before for many rickettsioses, the cutaneous damages at the inoculation site were histologically dominated by vasculitis and necrotic features (*11*). Rickettsial invasion of endothelial cells probably represents the first step of infection (*7*). Subsequent damage to the endothelium is followed by endothelial-cell activation and perivascular infiltration of lymphocytes, macrophages, and polymorphonuclear leukocytes, resulting in inflammatory vasculitis of dermal vessels, the histopathologic hallmark of rickettsial disease, and possibly thrombosis (*23*). However, in contrast with the other rickettsial diseases that are characterized by perivascular infiltration of T cells and macrophages, with some B lymphocytes and few neutrophils (*10**,**24**–**26*), the cutaneous damage of ATBF show vasculitis with polymorphonuclear leukocyte–rich inflammation. The predominance of neutrophils in inflammatory infiltrates may explain the importance of the local inflammation clinically observed at the site of inoculation, accompanied by the regional lymphadenitis.

By using immunohistochemical techniques, we demonstrated *R. africae* in cutaneous biopsy specimens from patients with ATBF. In accord with its obligate intracellular location, no extracellular organisms were observed in cutaneous biopsy specimens. Few bacterial antigens were found in vascular and perivascular locations within the cytoplasm of endothelial and inflammatory cells. In spite of the small amount of antigens detected, the inflammatory and necrotic cutaneous damage was histologically extensive. Our data indicate that *R. africae* replicates poorly in human tissues, likely because of its mild pathogenicity and the strong innate immune response. The local destruction of bacteria by the inflammatory reaction may explain the benign outcome of the disease.

In our study, immunohistochemical techniques had a sensitivity of 75%, a specificity of 100%, and a positive predictive value of 100%. Serologic tests, the most widely used diagnostic method for rickettsioses, had a sensitivity of 56% in early samples (*12*). Although our data suggested that immunohistochemical analysis might be more sensitive than serology in early samples, statistical analysis showed no significant difference between the 2 techniques. Regarding diagnostic methods applicable to skin biopsy specimens, immunochemical techniques were also more sensitive than culture (41%), which is restricted to specialized laboratories equipped with biohazard facilities; such techniques were also more sensitive than regular PCR (47%) (*27*). Moreover, the techniques exhibited a sensitivity similar to that of nested PCR (73.5%), previously found to be the most efficient diagnostic technique for spotted fever rickettsioses (*27*).

As Mediterranean spotted fever caused by *R. conorii* is endemic in the same regions of Africa as tick-bite fever, differentiation of the 2 syndromes by characterization of their etiologic agents may be useful for diagnostic and epidemiologic studies (*4**,**28**,**29*). The polymorphonuclear leukocyte–rich vasculitis, which dominates the histologic features of the inoculation eschar during ATBF, could suggest the diagnosis of this rickettsiosis but is not specific. The usual method for the diagnosis of rickettsioses is serologic testing. However, serologic cross-reactions are common among the rickettsiae in the spotted fever group, particularly between *R. africae* and *R. conorii* infections (*6*). Monoclonal antibodies had been developed to *R. africae* for use in assays to distinguish between *R. conorii* and *R. africae* in culture and skin biopsy samples (*20*). In this study, we used a monoclonal antibody produced in our laboratory to distinguish ATBF from Mediterranean spotted fever at the histologic level by immunohistologic methods. We presented the pathologic description of the first series of inoculation eschars from skin biopsy specimens of patients with ATBF. We showed that cutaneous damage is dominated by vasculitis, thrombosis, cutaneous necrosis, and a polymorphonuclear leukocyte–rich inflammatory reaction. Immunohistochemical detection of rickettsial antigens may be useful in diagnosing ATBF. Pathologists should now consider ATBF, a recently rediscovered rickettsiosis, during histologic analysis of inoculation eschars, especially in patients with a recent stay in sub-Saharan Africa.
